# Text Book of the Diseases of the Ear and Adjacent Organs

**Published:** 1884-10

**Authors:** 


TEXT-BOOK OF THE DISEASES OF THE EAR AND ADJACENT ORGANS. *By* DR. ADAM POLITZER. *Imperial-Royal Professor of Aural Therapeutics in the University of Vienua, Etc., Etc. Translated and Edited by* JAMES PATTERSON CAS-

sels, m. d., m. r. c. s., *Eng. Aural Surgeon to and Lecturer on Aural Surgery at the Glasgow Hospital and Dispensary for Diseases of the Ear. Cloth, pp. 800. Philadelphia:* Henry C. Lea's Son & Co.

This is the most voluminous work on the subject—a veritable encyclopedia of otology. The first and second numbers appeared in German in 1878–1882. The English edition forms one complete volume. The task of the translator has been performed in a conscientious and highly satisfactory manner. Dr. Cassels deserves much credit and the thanks of all his readers in enabling them to quench their thirst for knowledge at the very fountain head. B. B.

